# Impact and feasibility of a pharmacist-delivered pharmacotherapy rotation on pediatric resident education

**DOI:** 10.1080/10872981.2021.1955645

**Published:** 2021-08-04

**Authors:** Joseph M. LaRochelle, Aryn C. Karpinski, Bonnie Desselle, Sheila W. Chauvin

**Affiliations:** a Coleman Professor and Vice Chair, Division of Clinical and Administrative Sciences, Xavier University of Louisiana College of Pharmacy, New Orleans, LA, USA; b Clinical Professor of Pediatrics, Louisiana State University Health Sciences Center School of Medicine, New Orleans, LA, USA; c Kent State University, College of Education, Health, and Human Services, School of Foundations, Leadership, and Administration, Research, Measurement, and Statistics (RMS) Program, Kent, OH, USA; d Professor of Clinical Pediatrics, Chief Medical Education Officer, Leslie and Robert Suskind Vice Chair of Medical Education, Louisiana State University Health Sciences Center/ Children’s Hospital of New Orleans, New Orleans, LA, USA; e Professor Emerita, Department of Internal Medicine, Louisiana State University School of Medicine, New Orleans, LA, USA

**Keywords:** Pharmacist, medical resident, pharmacy, interprofessional education, pharmacotherapy

## Abstract

Pharmacotherapy training for pediatric residents is an important part of their overall education. Limited data exist describing formal engagement of clinical pharmacists in residency training. The objective of this study was to evaluate a novel pharmacotherapy rotation for learner gains and program feasibility. We designed a novel pharmacotherapy rotation (PTR) involving a pharmacist preceptor, pediatric resident, and final-year pharmacy students in the pediatric intensive care unit (PICU). Rotation objectives and content were based on learning gaps identified in a review of the resident curriculum. Data from PTRs completed 2014–2020 were used to evaluate PTR impact on residents’ knowledge and confidence in pharmacotherapy decision-making, and interprofessional valuing. We also addressed PTR feasibility for long-term and for adoption by others. Measures for demographic, knowledge, and confidence measures were administered to intervention and control groups. Measures for interprofessional valuing and post-PTR feedback were administered only to the intervention group. Pre-post gains were greater for intervention residents (n = 7) than for control (n = 10), (knowledge: p = 0.02, confidence: p < 0.0001). Interprofessional valuing gain for the intervention group was significant (p = 0.004). Few PTR changes have been necessary since initial implementation. Residents provided high ratings of PTR experiences and specific value-added benefits. Designing an inter-professional PTR within the existing PICU and pharmacy rotation enhanced feasibility, curriculum consistency, and flexibility to optimize inter-professional learning.

Participation in the PTR enhanced resident pharmacotherapy knowledge and decision-making, and engagement in interprofessional practice. Next steps include expanding the PTR to other settings and specialties with further evaluation study.

## Introduction

Pharmacotherapy education is essential to pediatric residents’ training, as 20% of the pediatric population uses at least one medication[1]. Limited evidence exists regarding pharmacotherapy education by clinical pharmacists [2,3]. Murphy et al. describes knowledge gained by family medicine residents as a result of a pharmacotherapy rotation[3]. In our day-to-day education of pediatric residents, we noticed gaps in their pharmacotherapy knowledge and confidence in drug decision-making. This prompted an in-depth review of pharmacotherapy topics and experiences throughout the resident curriculum and identified gaps formed the basis of a pharmacotherapy rotation (PTR). The PTR comprises a resident rotating directly with a pharmacist-preceptor, as part of the PICU team who engage in the drug decision-making process during daily patient care rounds. The PTR is supplemented by clinical pharmacist preceptor-resident reflective debriefing discussions who also engage in core content topic discussions with rotating senior pharmacy students that target pharmacotherapy treatment of various disease states.

We report educational program evaluation results that target the following: 1) effect of a two-week elective PTR in the PICU on resident knowledge and self-confidence in pharmacotherapy decision-making, and value for interprofessional team practice; and 2) feasibility for sustaining the PTR in the PICU and expanding it to other settings.

## Methods

This section describes methods for creating the PTR curriculum, followed by program evaluation methods. The PTR curriculum was co-developed by a clinical pharmacist and the pediatrics residency director (intensivist). Their review of the three-year residency program identified learning gaps for which PTR objectives and core content were established. Resident learning objectives of the PTR targeted enhancing their abilities as follows: 1) Develop a pharmaceutical care plan for acutely ill patients, 2) Increase confidence for making drug therapy management decisions based on changing clinical situations, 3) Discuss supporting evidence for pharmacotherapeutic decisions, and 4) Describe the clinical pharmacist role in the multidisciplinary care of pediatric critically ill patients.

Prior to initial implementation, a one-week pilot rotation with one pediatric resident was conducted to field test implementation and feasibility of the PTR (e.g., timing and logistical coordination of PTR activities within the existing PICU clinical and educational activities)[4]. The two-week elective PTR incorporated a single pediatric resident (max of two per PTR rotation) into an existing PICU pharmacy team consisting of the same pharmacist preceptor and two final year Doctor of Pharmacy students. In the PTR, the resident participated in the existing pharmacy team content discussions on pharmacotherapy treatment of various disease states, of which each discussion was expanded to include an extra 20 minutes to share basic pharmacist knowledge and address specific learning needs of the participating resident. In daily patient-centered interdisciplinary rounds, the resident served in the role of the clinical pharmacist, making suggestions to the PICU team regarding pharmacotherapy, guided by preparatory discussion with the pharmacist preceptor prior to rounds. PTR objectives were evaluated through daily preceptor observation of and interactions with the PTR resident.

The study design reflected educational program evaluation and received IRB approval for pursuing the following program evaluation questions (PEQ):

1. To what extent did PTR residents demonstrate increased knowledge and confidence in pharmacotherapy decision-making, compared to residents in the traditional PICU resident rotation (knowledge test, self-perceived pharmacotherapy decision-making confidence scale)?
2. To what extent did the PTR experience change participating residents’ value for interprofessional practice (Interprofessional Socialization and Valuing Scale)?
3. To what extent was the PTR implemented and sustained as intended (process evaluation, post-PTR resident questionnaire responses, and mid-course adjustments)?
4. To what extent is the PTR a feasible option for long-term continuation in the PICU setting and adoption/adaptation in other settings (formative and summative evaluation, post-PTR resident questionnaire, reflection on adoption and implementation experiences)?

Participation in the evaluation study was voluntary and had no impact on residents’ performance evaluation or academic status. Data collection occurred 1 September 2014 through 10 February 2020. Each participant followed specific instructions to consistently re-create a unique record code when completing a study measure to facilitate matching responses over time and measures, while maintaining anonymity. Open to all 2^nd^ and 3^rd^ year residents, those who completed the PTR elective, comprised the intervention group. The control group included all other residents who completed the existing PICU resident rotation during the same period. The residents in the control group learned pharmacotherapy during existing direct patient care activities of the resident PICU rotation. They did not participate in the clinical preceptor-facilitated PTR topic discussions, patient-debriefing discussions or development of patient-specific pharmacotherapeutic plans.

A demographic questionnaire was administered to control and intervention groups that included level of training, previous ICU rotations, age, gender. Marital and parental status questions were also included, because prior research suggests higher confidence in skills when residents are married or have children[5]. Several measures were administered, as described below.

For pharmacotherapy knowledge (PEQ 1), a 19-item, case-based, multiple-choice knowledge test was administered at the beginning and end of rotations to control and intervention groups that included basic pharmacokinetic differences between neonates, children, and adolescents, along with proper drug selection, dosing, and monitoring for sepsis, sedation and analgesia, and antibiotics. The test was developed by the principal investigator/clinical pharmacist, with expert review by two experienced clinical PICU pharmacists, a psychometrician, the residency program director (pediatric intensivist), and two other pediatric intensivists.

Using the same method for creating the knowledge test, a measure was developed to assess self-perceived confidence in pharmacotherapy decision-making (PEQ 1), as no existing measures were identified. A nine-item scale using a six-point, Likert-type response scale (Appendix A) was administered at the beginning and end of rotations to intervention and control groups.

For PEQ 2, the Interprofessional Socialization and Valuing Scale (ISVS), a copyrighted instrument, used with author permission, was administered at the beginning and end of rotations to only the intervention group[6]. The ISVS is a 24-item measure using a seven-point Likert-type scale (1 = not at all to 7 = to a very great extent) to assess self-perceived value, comfort, and ability to work in an interprofessional team[6]. The ISVS has demonstrated good internal consistency and reliability in previous studies involving health-care professions, including physicians (Total scale Cronbach’s alpha = 0.86–0.92; subscale scores = 0.79–0.89) [6,7].

At the end of rotations, the intervention group also completed a 5-item post-PTR questionnaire to rate perceived contributions of the PTR activities to their development as a physician (1 = definitely no to 6 = definitely yes). Responses to two open-ended questions provided qualitative feedback regarding most beneficial experiences and suggestions for improvements. Residents’ responses to this questionnaire, informal resident feedback, and day-to-day observations contributed to answering PEQ 3 and 4.

Data screening, including analysis of skewness, and calculation of descriptive statistics occurred prior to comparative analyses. Descriptive statistics for the demographic information, independent t-tests to compare intervention and control groups, and paired t-tests for pre-post within-group comparisons were completed. Statistical methods were supported by Norman (2020) for Likert-type data and de Winter (2013) regarding very small sample sizes [8,9].

## Results

A total of 36 residents (10 intervention, 26 control) participated in the study. Complete and usable data (pre and post measures) were obtained from seven of 10 (70%) in the intervention group. For the control group, post-rotation data collection was logistically difficult, given residents’ changes in location and schedule of subsequent rotations. Consequently, complete data (pre and post measures) for 10 of the 26 residents in the control group (38.5%) were available for data analysis.

Table 1 summarizes demographic characteristics for intervention and control groups. Age and gender characteristics were similar between the two groups. The intervention group consisted of third-year residents; whereas the control group included mostly second-year and some third-year residents (p = 0.02). In the pediatrics residency curriculum, all residents complete at least one PICU rotation prior to entering the third year. The intervention group included less married residents, but more with children than in the control group.

**Table 1.** Descriptive demographic statistics for intervention (n = 7) and control (n = 10) groups

Group	Item	Mean	Standard Deviation	Min	Max
Control group	Level*	2.23	0.50	2	3
	Age	28.81	2.95	27	30
	ICU Rotation**	1.14	1.21	0	3
	Sex Male Female	Number (%) 1 (10%) 8 (80%)			
	Married	Number (%) 7 (70%)			
	Children	Number (%) 1 (10%)			
Intervention group	Level*	2.89	0	3	3
	Age	28.8	0.98	28	30
	ICU Rotation**	2.6	0.85	2	4
	Sex Male Female	Number (%) 1 (14%) 6 (86%)			
	Married	Number (%) 2 (29%)			
	Children	Number (%) 2 (29%)			

*Resident Level; Independent t test, p-value = 0.02, p set at 0.05, significant difference.

** ICU Rotation; Independent t test, p-value = 0.004, p set at 0.05, significant difference

Calculation of Cronbach’s alpha coefficients produced the following results: Confidence measure = 0.91, ISVS total score = 0.83, and for subscale scores were Comfort = 0.67, Ability = 0.75, and Value = 0.88. Below are results by each program evaluation question (PEQ) listed in Methods.

Regarding PEQ 1 (knowledge, confidence), Table 2 summarizes statistical results of pre-post scores for intervention and control groups. No statistically significant differences between intervention and control groups were observed in pre-rotation knowledge and confidence scores (p = 0.09 and p = 0.48, respectively). Statistically significant differences were observed between groups for post-rotation knowledge and confidence (p = 0.01, p = 0.002, respectively), with higher scores for the intervention group. Pre-post knowledge and confidence total score differences were not statistically significant for the control group, but statistically significant for both scores in the intervention group.

**Table 2.** Summary results for knowledge tests and confidence questionnaire in intervention (n = 7) and control groups (n = 10)

	Knowledge Mean (SD)			Confidence Mean (SD)		
Variable:	Pre	Post	p	Pre	Post	p
Control	10.70 (3.3)	10.44 (4.1)	0.15	4.71 (0.6)	4.92 (0.6)	0.17
Intervention	12.86 (1.5)	14.29 (2.0)	0.02*	4.70 (0.6)	5.65 (0.4)	**0.00009**
p	0.089	**0.012**		0.48	**0.0016**	

*Independent t tests for comparisons between intervention and control groups. Paired t tests for pre-post difference within group comparisons. Alpha set at p ≤ 0.05. P values shown in bold are statistically significant after Bonferroni adjustment calculation.

For PEQ 2 (interprofessional value), the results summarized in Table 3 reveal statistically significant pre-to-post gains for total score and for each subscale score (total p = 0.004; ability p = 0.006; value p = 0.005; comfort p = 0.005).

**Table 3.** Summary results for ISVS in Intervention group (n = 7)

ISVS Scale	Pre-rotation Mean (SD)	Post-rotation Mean (SD)	p
Total Scale	137.3 (10.8)	159.7 (8.1)	**0.004**
Ability Sub-scale	52.7 (3.9)	60.7 (3.0)	**0.006**
Value Sub-scale	53.1 (4.5)	61.1 (2.5)	**0.005**
Comfort Sub-scale	31.4 (3.4)	37.9 (3.4)	**0.005**

*Paired t test for pre-post difference within group comparisons. Alpha set at p ≤ 0.5. P values show in bold are statistically significant after Bonferroni adjustment calculation.

Results for PEQ 3 (process evaluation) demonstrated that implementation of the two-week PTR required only a few, minor process adjustments at the onset. For example, foundational topic discussions (e.g., pharmacist training, basic pharmacokinetic principles) occurred at the beginning of the rotation, but remaining topics occurred when prompted by actual PICU patients. Based on resident feedback, core content discussions for common patient problems were moved to the beginning of the rotation, allowing residents more time to read and prepare in advance for these discussions. During the PTRs, additional topic discussions emerged to address each resident’s specific goals and learning needs. Debriefing discussions occurred daily between the resident and the pharmacist preceptor to elicit continual feedback throughout the rotation.

Residents’ responses to the 5-item post-PTR questionnaire consistently reflected positive impact on their physician development and value-added features supporting sustainability. For example, residents reported that assuming the role of pharmacist on the team was particularly helpful to their physician development and future effectiveness for working with clinical pharmacists. Frequently, questionnaire responses reflected specific benefits of learning with and from pharmacy students. Results also revealed the PTR residents most valued the patient-specific debriefing discussions with the pharmacist preceptor . Highest rated PTR activities were preparatory reading for content discussions with the pharmacy students (item mean score = 5.7/6), followed by learning in patient rounds and bedside teaching (during which they assumed the clinical pharmacist role) (5.3/6).

For PEQ 4 (feasibility, sustainability), effective continuation and increasing receptivity by learners and educators support its feasibility and sustainability. From first conception, design strategies targeted the threat of increased workload for residents, PICU team, and pharmacist preceptor. Unsolicited anecdotal feedback from residents during the PTR consistently reflected that their perceptions of workload was manageable and no more than other rotations. Additional time commitments were negligible for the PICU team and the pharmacist preceptor, since PTR activities were strategically situated within existing PICU educational and clinical activities. Additional time and effort were necessary only initially for the residency program director and clinical pharmacist involved in the initial curriculum mapping and PTR planning processes. Importantly, the implementation of the PTR did not require any additional financial or personnel resources.

Active engagement of the PTR resident was embraced by the attending-led PICU team because these residents, acting in the role of clinical pharmacist, added benefit and value to team processes, particularly for pharmacotherapy recommendations and decision-making. Reciprocally, the PTR residents benefited from additional practical application and collaboration with PICU team members regarding pharmacotherapy topics, issues, and decisions. During the study period, we learned that upon completing the PTR, some residents pursued additional rotations with pharmacists in other settings. Also, residents in other programs (e.g., Pediatrics/Emergency Medicine) asked to participate, after hearing about or witnessing the PTR in action. A separate investigation examining the PTR from the Pharm D student perspective showed improved interprofessional socialization and valuing and comfort in interacting with physicians (manuscript pending publication).

As the PTR implementation progressed, the following enhancements to the overall pharmacotherapy curriculum for all residents in the institution resulted: 1) pharmacist engagement was added to attending physician-run general topic resident discussions that occurred throughout a regular rotation in the PICU (i.e., control group), 2) time for pharmacist-led education increased during patient-care rounds in the PICU, and 3) pharmacist-led sessions increased in regular resident education conferences for all-year residents and medical students in the institution. Since implementation of the PTR, additional clinical pharmacists have been hired and the PTR innovation has been expanded into other settings, such as cardiac and neonatal intensive care, and antimicrobial stewardship. Consequently, this observed spread/diffusion of PTR features incorporated into these other educational activities suggest positive institutional effects beyond PTR residents’ educational and professional development gains. Observations and informal interactions with educators/leaders indicate interest and efforts to adopt/adapt the PTR for other pediatric settings, as clinical pharmacy services are expanded.

## Discussion

Results of our PTR implementation and evaluation are important to thinking and practice in resident education for several reasons. First, pharmacotherapy education is essential to pediatric resident training, especially since medications are a common element of caring for their patients and few examples of such education exist in the literature [1,3]. This report provides one effective and feasible innovation that could be adopted, adapted, or used to stimulate creative learning strategies more broadly for enhancing pharmacotherapy decision-making and management in pediatric patient care, and potentially in other specialties.

Second, the PTR sought to resolve specific pharmacotherapy knowledge gaps through a real-life, practice-based, inter-professional rotation that utilized existing resources, structure, and processes within the PICU setting, without substantial increases in time, effort, personnel, or finances. Implementation results support feasibility and long-term sustainability.

Third, as demonstrated in the PTR innovation, educational change often occurs by evolution. The PTR implementation stimulated further inquiry into the change process literature; Rogers’ model for diffusion of innovation provides a useful framework within which to situate our PTR efforts, particularly in terms of Rogers’ specific features of an innovation (e.g., PTR) and of the organizational context to facilitate successful adoption, implementation, and sustainability[10]. For example, using Rogers’ features of an innovation, reinforced our approach to creating the PTR to address specific knowledge and application gaps, and as such, facilitated visibility of its particular *relative advantage* over the traditional pharmacotherapy education in the PICU. The PTR design also maximized its *compatibility* with existing PICU structures and processes and minimized its *complexity* to facilitate ease of implementation and sustainability. Finally, because the PTR occurred within the PICU and alongside other educational and clinical activities, the *observability* and *trialability* of the PTR by others was enhanced; thus, facilitating receptivity, adoption, implementation, and ultimately, sustainability and incorporation of the PTR into everyday practice. Similarly, Rogers’ organizational features were optimized to facilitate successful change through facilitating increased *visibility of leadership*, effective use of existing *social systems* and *communication channels* to engage individuals, and effective management of the *pace* at which change occurred. Essentially, our efforts to design features of the PTR to fit well within the existing rhythm of education and patient care in the PICU enhanced its implementation and effectiveness, and contributed to its spread/diffusion other potential clinical education settings. Perhaps our PTR design and results and the use of Roger’s diffusion of innovation model, specifically through optimizing features of the innovation and of the organization, may facilitate innovative thinking and successful innovation in other settings and specialties.

Fourth, and specific to inter-professional learning and practice, the increase in PTR residents’ value for inter-professional practice after only a two-week, inter-professional rotation was encouraging. For example, in this study, the ISVS mean total scale score was 137 for pre-rotation (132 was reported in King’s original study) and the post-PTR mean total scale score was 160 (maximum possible total scale score is 168)[6].

Often the case with educational innovations, small sample size can be a study limitation. While our study reflected low numbers of participants in both groups, results reflected some statistically and educationally significant findings. A larger or expanded sample or additional replication of the PTR implementation and evaluation at other sites could enhance generalizability and suggest future enhancements. In addition, residents’ participation in the PTR elective was voluntary. Consequently, participants may have been particularly interested and motivated to enhance their abilities in this area. Another limitation was a lack of long-term follow-up measurement of PTR resident gains and impact on subsequent everyday practice.

## Conclusions

While results of this work are encouraging, additional study is needed. Implementation with other clinical pharmacist preceptors and in other settings/specialties would enhance understanding and insight. Results suggest that if the PTR was integrated earlier in resident training, then one might expect the same or greater benefits as reported here. How much more time and for what additional gains and benefits? And what about in other specialties? Since the curriculum mapping process, general rotation design, and the confidence and ISVS measures are not specific to pediatrics, the PTR innovation might be applicable to other residency and fellowship programs to enhance pharmacotherapy education within similarly existing structures and processes.

## Practice points

1. Careful analyses of resident curriculum using different lens (e.g., pharmacotherapy) can reveal important learning gaps for explicit workplace-based learning and practice.
2. Enhancing compatibility of an educational innovation with existing environmental structures and processes facilitates successful, long-standing change, even in highly complex settings.
3. Meaningful educational change can be accomplished without the need for additional time or money.
4. Shared learning and real-life practice between pharmacy and medicine near-peer learners facilitates receptivity, ability, and value for inter-professional education and practice.
5. Expansion of the PTR to other clinical settings is feasible with minimal adaptation and will contribute to increased rigor in program evaluation.

## Appendix A

**Self-Perceived Confidence in Pharmacotherapy Decision-Making Questionnaire**

**DIRECTIONS**: For each item, mark the one response that best reflects your view.
